# Advances in the Critical Care Management of Ischemic Stroke

**DOI:** 10.1155/2013/510481

**Published:** 2013-05-16

**Authors:** Vineeta Singh, Nancy J. Edwards

**Affiliations:** Department of Neurology, University of California-San Francisco, San Francisco General Hospital, 1001 Potrero Avenue, San Francisco, CA 94110, USA

## Abstract

Given recent advances in diagnostic modalities and revascularization capabilities, clinicians are not only able to rapidly and accurately identify acute ischemic stroke, but may also be able to aggressively intervene to minimize the extent of infarction. In those cases where revascularization cannot occur and/or the extent of infarction is large, there are multiple strategies to prevent secondary decompensation as the stroke evolves, for instance, if malignant cerebral edema should develop. In this paper, we will review the indications for specialized ICU care for an ischemic stroke patient, the treatment principles, and strategies employed by neurointensivists to minimize secondary neuronal injury, the literature in support of such strategies (and the questions to be addressed by future studies), all with the ultimate goal of increasing the likelihood of favorable neurologic outcomes in our ischemic stroke population.

## 1. Introduction

There has been considerable evolution in the treatment of acute ischemic stroke in recent years,including progressive improvements in our ability to revascularize patients and in aggressive therapies to decrease secondary brain injury (such as early decompressive hemicraniectomy for large hemispheric strokes). As such, the role of the neurointensivist in the care of ischemic stroke patients has evolved as well. Approximately 15 to 20% of ischemic stroke patients will require care in an intensive care unit (ICU) [1]—this includes patients at considerable risk of hemorrhagic transformation or the development of malignant cerebral edema, patients who require intubation due to brainstem stroke or a decline in the level of alertness, and patients exhibiting hemodynamic instability ranging from atrial fibrillation with rapid ventricular rate to symptomatic hypotension with extension of infarction. And, as studies of other groups of critically ill neurologic patients have suggested [2], the care of unstable ischemic stroke patients in a neurosciences ICU staffed by trained neurointensivists results not only in greater efficiency of care, but also in improved patient outcomes. In one retrospective study by Bershad et al., critically ill ischemic stroke patients treated by a dedicated neurointensivist team not only had shorter stays in the ICU and in the hospital in general, but also a greater likelihood of being discharged to home [3]. As a result, the Joint Commission's 2011 proposed requirements for comprehensive stroke center certification included the recommendation for “an intensive care unit for complex stroke patients that includes staff and licensed independent practitioners with the expertise and experience to provide neurocritical care.” In this paper, we will discuss the indications for admission of an ischemic stroke patient to a neurosciences ICU; we will also examine the treatment principles and strategies employed by neurointensivists in an effort to increase the proportion of patients with favorable neurologic outcomes.

## 2. Postthrombolysis and/or Intra-Arterial Revascularization

Within the past decade or so, advances in acute revascularization have truly transformed the care of patients presenting with ischemic stroke. Intravenous tissue-type plasminogen activator (tPA) is of proven and substantial benefit for selecting patients with acute cerebral ischemia. Patients with occlusions of large intracranial arteries may also undergo intervention with intra-arterially deployed devices (such as the recently developed stent retrievers) in an effort to achieve rapid and effective vessel recanalization, although the role of these devices is uncertain at present given recently published trials suggesting that the functional outcomes are not improved with further intra-arterial therapy compared to intravenous tPA alone [4, 5]. 

Nevertheless, these aggressive revascularization strategies are not without risk—specifically, the risk of life-threatening intracranial hemorrhage. Thrombolysis with intravenous tPA is associated with symptomatic intracranial hemorrhage (sICH) in approximately 3 to 6% of patients [6–8]. For this reason, patients who have received intravenous tPA, intra-arterial tPA, or intra-arterial embolectomy require intensive neurologic monitoring in an ICU or a dedicated stroke sciences unit for at least 24 hours. To minimize the risk of hemorrhagic transformation in this particularly vulnerable population, blood pressure should be frequently monitored, and elevated pressures must be treated accordingly. Specifically, systolic pressures should be less than 180 mm Hg and diastolic pressures less than 105 mm Hg for the first 24 hours after thrombolysis [9]. As stroke patients are often hypertensive (even those without a history of hypertension may be hypertensive in the acute stroke period), intravenous agents may be required—Labetalol (10 mg IV every 10–15 minutes) or even a Nicardipine infusion (2.5–5 mg/hr; titrated to a maximum of 15 mg/hr) is frequently used for strict blood pressure control.

If a patient neurologically deteriorates during or shortly after the infusion of intravenous (or intra-arterial) tPA, emergent neuroimaging should be obtained to determine if hemorrhagic transformation has occurred. In the case of sICH, a type and cross, prothrombin time/partial thromboplastin time, platelet count, and fibrinogen level should be drawn. Reversal agents should be considered; though protocols vary, fresh frozen plasma (two units every 6 hours for 24 hours), cryoprecipitate (10–20 units), and platelets (4–8 units) are often transfused [10]. If these agents fail, the antifibrinolytic aminocaproic acid (Amicar) may be considered (5 grams in 250 mL NS). And, as with spontaneous intracranial hemorrhage, therapies to reduce intracranial pressure (mannitol or hypertonic saline) or even neurosurgical decompression (posterior fossa hemorrhage, lobar/superficial hemorrhage refractory to medical therapy) may be required.

 Serious systemic hemorrhages after revascularization are rare, although if undetected, may also be life-threatening. Interventional procedures begin with the cannulation of the femoral artery. Occasionally, hemorrhage from the cannulation site, including severe retroperitoneal hemorrhage, may occur. Such bleeding may be intensified if intravenous tPA was administered as a bridge to intra-arterial therapies. In these cases, manual compression of the groin site in addition to volume replacement with red cells, platelets, and fresh frozen plasma may be required. Finally, the administration of tPA can result in one other serious complication—the development of orolingual angioedema. Orolingual angioedema reportedly occurs in 1 to 5% of patients; the angioedema is typically mild, transient, and contralateral to the ischemic hemisphere [11]. If severe enough, the angioedema may result in partial airway obstruction. Intravenous antihistamines, corticosteroids, and histamine type 2 receptor antagonists are often given; patients who develop frank stridor should be intubated. 

## 3. Large Hemispheric Stroke and Malignant Cerebral Edema

 Patients with large hemispheric stroke often deteriorate neurologically and are therefore frequently managed in the intensive care unit. Symptomatic hemorrhagic transformation and the evolution of cerebral edema are the primary causes of deterioration. Classically, cerebral edema peaks 2 to 5 days after the onset of infarction [12]. A subset of patients with large hemispheric stroke, primarily those with complete middle cerebral artery (MCA) territory infarction, will dramatically deteriorate within the first 24 to 48 hours, with evidence of massive edema, severe midline shift, and compression of the basal cisterns on neuroimaging. These “malignant” MCA infarctions constitute 1 to 10% of all supratentorial ischemic strokes, and, historically, mortality is considerably high, ranging from 50 to 80% [13, 14]. Early identification of patients likely to develop malignant cerebral edema is essential as certain therapies (such as decompressive hemicraniectomy) are particularly helpful if performed early, prior to complete neurologic deterioration and herniation. The NIH Stroke Scale (NIHSS) score often surpasses 16–20 if the dominant hemisphere is involved, 15–18 in malignant infarctions of the nondominant hemisphere. Radiologic predictors of malignant cerebral edema include (1) early hypodensity greater than 50% of the MCA territory on CT or diffusion lesion volume greater than 82 mL within 6 hours of stroke onset, and (2) involvement of adjacent vascular territories such as the anterior cerebral artery (ACA) or posterior cerebral artery territories [15].

There has been considerable interest within the past decade in surgical decompression for those patients with large hemispheric stroke and malignant cerebral edema. The primary goal of decompression (hemicraniectomy and duraplasty) is to give edematous tissue space to expand outside the cranial vault, reducing tissue shifts and pressure within the intracranial compartment, thereby restoring cerebral perfusion and minimizing derangements in oxygenation of noninfarcted tissue. In 2007, a pooled analysis of three European randomized controlled trials (DECIMAL, HAMLET, and DESTINY) compared decompressive hemicraniectomy with best medical management in patients from 18 to 60 years old with an NIHSS score greater than 15, decreased level of consciousness without bilaterally fixed/dilated pupils, with a hypodensity involving at least 50% of the MCA territory on CT [16]. For this pooled analysis, a maximum time window of 48 hours from stroke onset to surgical decompression was adopted. The case fatality rate was substantially lower in the surgical decompression group (28% versus 78% in the conservative arm), with an absolute risk reduction of 50%. With regards to functional outcome, decompression improved the odds of a favorable one, defined in the analysis as a modified Rankin Scale (mRS) score of 1 to 4 (75% versus 24%). However, whether to interpret a mRS score of 4 (moderately severe disability, unable to walk, or attend to one's own bodily needs without assistance) as favorable is a question best answered in conjunction with those closest to the individual patient. There is also considerable debate regarding surgical decompression in patients older than 60, although this represents approximately 40 to 50% of the patients with malignant MCA infarction [17]. DESTINY II is a large randomized controlled trial of patients 61 and older to specifically address whether these patients may benefit from surgical decompression, particularly in terms of functional outcome.

The other approach to cerebral edema and elevated intracranial pressure involves the use of hyperosmolar agents such as mannitol and hypertonic saline. Essentially, both mannitol and hypertonic saline work by the formation of a relatively hypertonic intravascular space; this promotes the osmotic flow of water outward from the brain parenchyma. Mannitol is often administered as a bolus in doses of 0.25 to 1.0 g/kg every 4 to 6 hours, whereas hypertonic saline may be administered as a bolus (23.4%) in roughly equiosmolar doses to mannitol or as a continuous infusion (3%). One small prospective trial by Schwarz et al. investigated the use of mannitol versus hypertonic saline (7.5% hypertonic saline hydroxyethyl starch) specifically in ischemic stroke patients. 16 of 16 “episodes,” defined as an ICP greater than 25 mm Hg or the development of pupillary abnormalities, responded to hypertonic, saline whereas 10 of 14 episodes responded to mannitol. The mean ICP reduction was 11 mm Hg with hypertonic saline, 5 mm Hg with mannitol [18]. Kamel et al. conducted a meta-analysis in 2011 of the five prospective trials comparing hypertonic saline with mannitol; 3 of these trials included stroke patients. ICP was successfully reduced 78% of the time with mannitol, 93% of the time with hypertonic saline [19]. A large scale, prospective, blinded study using equiosmolar doses and assessing functional outcomes is essential to determine if a true comparative benefit exists with either of these agents. In addition, no studies have addressed the prophylactic use of mannitol or hypertonic saline to reduce edema and tissue shifts prior to the onset of intracranial hypertension.

In the future, we may be able to prevent the development of malignant cerebral edema entirely. Within the past decade, experimental models of ischemic stroke have been used to identify the cellular mediators responsible for cytotoxic edema formation. One such mediator is a nonselective cation channel regulated by sulfonylurea receptor 1 (SUR1). Blockade of SUR1 by glibenclamide (glyburide) has been demonstrated to reduce the formation of cytotoxic edema, infarct volume, and mortality by approximately 50% in a mouse model of MCA occlusion [20]. In fact, in one study of severe MCA ischemia/reperfusion in rats, glibenclamide administered during the first 6 hours after ischemia was as effective as the use of early decompressive craniectomy in preventing death from malignant cerebral edema [21]. Recently, a prospective, open label Phase IIa trial of RP 1127—an intravenous formulation of glyburide—was completed in 10 patients with severe anterior circulation ischemic strokes (mean infarct volume 82 mL; mean NIHSS score 19). Malignant cerebral edema requiring the use of hyperosmolar agents or decompressive craniectomy occurred in only 2 of the 10 patients; 9 of the 10 patients had a 30-day mRS score of greater than or equal to 4 [22]. A larger, multicenter, randomized controlled trial comparing RP 1127 to placebo in patients with severe anterior circulation ischemic stroke, the GAMES trial, has recently begun. 

## 4. Blood Pressure—General Parameters, Augmentation via Vasopressors

Hypertension in the acute stroke period is frequent. Elevated blood pressures are documented in approximately 80% of patients, including those without a history of hypertension. Severe arterial hypertension may aggravate cerebral edema and contribute to hemorrhagic transformation of infarcted tissue. Conversely, severe arterial hypotension is also detrimental, as rapid reductions in mean arterial and cerebral perfusion pressures (CPPs) may extend areas of ischemia. Studies suggest that even relative hypotension may be an independent predictor of poor outcomes in ischemic stroke patients [23]. In the SCAST trial, 2029 acute stroke patients with a mean blood pressure of 171/90 mm Hg were randomized to candesartan versus placebo during the first 7 days after stroke [24]. During this period, blood pressures were significantly lower in the candesartan group (mean blood pressure 147/82 mm Hg versus 152/84 mm Hg in the placebo group). Interestingly, analysis of 6 months functional outcomes suggested a higher risk of poor outcomes in the candesartan-treated group (adjusted odds ratio 1.17). As such, blood pressures are generally permissive in the first several days after stroke, with a systolic pressure threshold of 220 mm Hg and a diastolic pressure threshold of 120 mm Hg during the first 24 hours as per AHA/ASA guidelines [9]. Pressures may require earlier reduction, though, if patients develop angina/myocardial infarction, significant pulmonary edema, or renal dysfunction.

**Figure 1:**
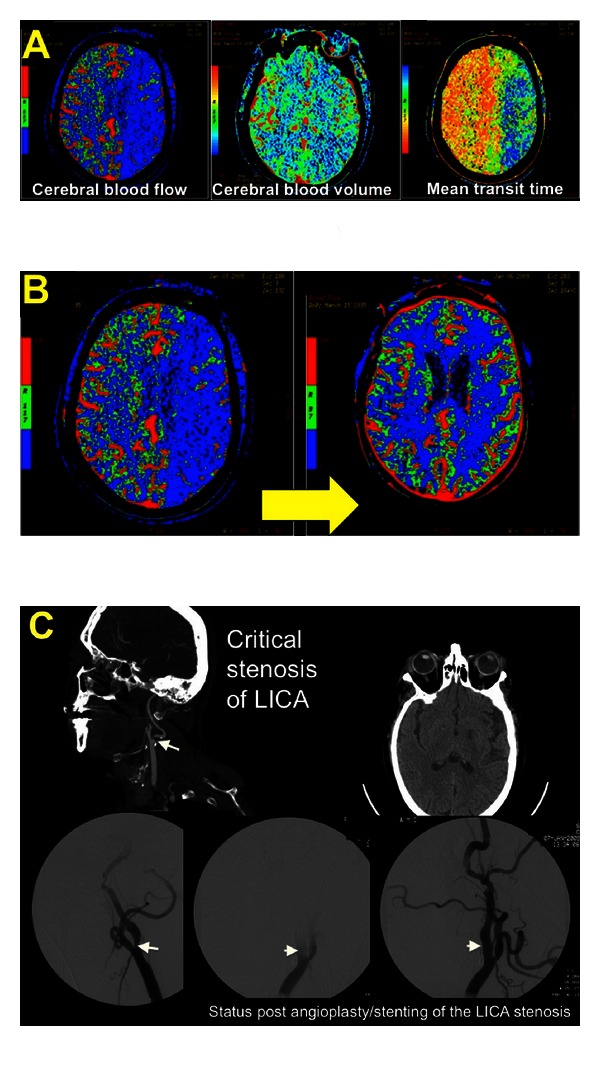
A 73 year-old woman underwent intramedullary fixation of a fractured right femur. She received 2 doses of Hydralazine for elevated BPs the evening after surgery, resulting in a decline in her SBP from 190 mm Hg to 110 mm Hg. The following morning, she was noted to be confused, nonverbal, and paretic in her right arm. An inpatient stroke code was called, and a CT/CTA/CTP was obtained. The perfusion imaging revealed a prolonged MTT, decreased CBF, and relatively preserved CBV within the left hemisphere suggestive of ischemia within this territory (A). She was transferred to the ICU for further management. (B) diagrams the resolution of these perfusion deficits with a marked improvement in CBF following aggressive intravenous fluid resuscitation, blood transfusion for anemia, and BP augmentation with pressors. This was accompanied by a significant clinical improvement as well. Her CTA neck revealed the etiology of her stroke to be a critical stenosis of her left internal carotid artery (C); she underwent angioplasty and stent placement and ultimately had minimal residual symptoms in the form of naming and paraphasic errors. There was no hemorrhagic transformation post-induced hypertension or postprocedurally.

On the other hand, there are certain stroke patients who may benefit from augmentation of blood pressure via the administration of isotonic fluids or even vasopressors. For instance, patients with severe stenosis of the carotid or basilar artery may develop fluctuating symptoms if relatively hypotensive (Figure 1). In 2001, Rordorf et al. published the results of one prospective trial investigating the induction of hypertension in a series of acute stroke patients. Neosynephrine was administered to augment systolic pressures to 160 or by at least 20%; responders were those patients demonstrating neurologic improvement (NIHSS score increase of at least 2 points) with higher pressures. 7 of 13 patients were considered responders—86% of these responders had evidence of very severe carotid stenosis or occlusion of the MCA stem on cerebrovascular imaging [25]. No adverse events were documented throughout the period of induced hypertension. Larger trials are required to delineate whether induced hypertension significantly improves functional outcomes and to confirm the safety of pressor administration in stroke patients—particularly those after thrombolysis or those with suboptimal endovascular recanalization. 

## 5. Advanced Neuromonitoring?

Advanced neuromonitors—those devices able to quantitatively and continuously measure intracranial pressure, brain temperature, brain tissue oxygenation, oxygen saturation of the jugular vein, and even the biochemical milieu of the cerebral interstitium—are increasingly being used for patients with severe central nervous system injury such as traumatic brain injury and subarachnoid hemorrhage. Theoretically, these monitors signal the presence of derangements capable of causing secondary neuronal injury (and poorer functional outcomes) if left unchecked. The question begs could these monitors be helpful in patients likely to deteriorate from ischemic stroke? In the ischemic stroke population, it is presently unclear whether these monitors add helpful information above and beyond that provided by a relatively crude yet entirely noninvasive monitor—the neurological examination. For instance, in one recently published randomized controlled trial of intracranial pressure monitoring (ICP) in patients with traumatic brain injury, functional outcomes were not superior in those cared for with an ICP monitor (the target being an ICP of less than 20 mm Hg) compared to those whose treatment was guided by the neurological examination and neuroimaging without any advanced monitors [26]. In regards to ICP monitoring in patients with large hemispheric stroke, patients often deteriorate clinically without evidence of global ICP elevation or CPP reduction [27]. Schwab et al. reported intracranial hypertension (defined as an ICP greater than 15 mm Hg) in only 26% of their patients with large hemispheric stroke; CPP was reduced (less than 55) in only 11% [28]. Indeed, those patients who clinically herniate often do so without an elevation in measured ICP. And, for those patients with malignant infarction who undergo decompressive hemicraniectomy, absolute ICP measurements are likely less reliable (although the general trend of ICP measurements may be valid). 

There is even less data on the subject of continuous jugular venous oximetry in ischemic stroke patients. Theoretically, measurement of venous oxygen saturation in the jugular bulb (SjvO_2_) provides information regarding global cerebral oxygen metabolism. In one study of SjvO_2_ monitors in 10 patients with large hemispheric stroke, of the 101 measurements obtained, only 2 were less than 50%, the threshold suggestive of secondary ischemia (at least in traumatic brain injury patients). These patients also had cerebral blood flow (CBF) probes inserted for frequent CBF measurements; CBF was substantially decreased without concurrent reductions in SjvO_2_ on 19 occasions [29]. This study emphasizes cautious interpretation of global oxygen monitors in patients with large, focal, fixed lesions such as hemispheric infarction. Oxygen extraction is likely minimal in infarcted tissues and may result in falsely reassuring jugular saturations unless the remainder of the parenchyma is dramatically ischemic. 

## 6. Prevention of Secondary Neuronal Injury—Glucose and Temperature Control

Hyperglycemia is quite frequent in patients presenting with acute ischemic stroke, even in nondiabetics. Large infarcts and those involving the insular cortex in particular predispose to hyperglycemia. In experimental models, hyperglycemia provokes metabolic demand in the ischemic penumbra; lactic acid and various free radicals are liberated, resulting in neuronal cell lysis and/or degradation of the blood-brain barrier [30]. Numerous studies have reported several negative outcomes in hyperglycemic stroke patients, including higher rates of hemorrhagic transformation, larger volumes of cerebral edema, and greater odds of disability and death [31]. It is therefore reasonable to try and reduce significantly elevated serum glucose levels, although there is considerable controversy in how to do so. In 2009, the results of the NICE-SUGAR study were published; this large, international, randomized controlled trial compared intensive glucose control (target glucose 81–108) with conventional glucose control (180 or less) in medical and surgical ICU patients. Surprisingly, intensive glucose control increased the absolute risk of death at 90 days by 2.6 percentage points, representative of a number needed to harm of 38 [32]. In addition, severe hypoglycemia (less than 40) was recorded in 6.8% of intensive glucose control patients versus 0.5% of conventional control patients. To further investigate optimal glucose control in acute ischemic stroke patients specifically, the NINDS is currently sponsoring the SHINE trial—a multicenter, randomized trial evaluating whether glucose control with intravenous insulin (target glucose 80–130) will result in improved functional outcomes in acute ischemic stroke patients. At present, the AHA/ASA and European Stroke Initiative guidelines recommend a target glucose of less than 140–180 mg/dL.

Temperature regulation in the neurosciences ICU is becoming essential, particularly as the goal may extend beyond fever control to hypothermia. Fever, akin to hyperglycemia, causes secondary neuronal injury; as such, the target temperature for patients with acute central nervous system injury of any etiology should be normothermia (36 to 37 degrees Celsius), at the very least. Hypothermia for neuroprotection in patients with global cerebral hypoxia/ischemia secondary to cardiac arrest is fairly routine; in the future, hypothermia as a neuroprotectant may be extended to acute ischemic stroke patients. Hypothermia is also considered in those with medically refractory elevations in ICP. Whether the goal is normothermia or hypothermia, temperature control can be challenging, particularly in stroke patients who are awake. Antipyretics such as Tylenol are often ineffective, and surface cooling (even endovascular cooling) can be quite uncomfortable and often prompts shivering. Several institutions have generated “antishivering protocols”—buspirone, meperidine, and cutaneous counterwarming are often firstline measures; opiates and dexmedetomidine may be useful if these fail [33]. If shivering continues, sedation with high-dose propofol or even neuromuscular blockade may be required.

In conclusion, the outcomes of devastating neurological emergencies such as acute ischemic stroke may be measurably improved by treatment in a dedicated neurosciences intensive care unit utilizing the principles and strategies outlined in this paper. Multiple trials currently underway will hopefully further define care of the ischemic stroke patient and result in superior functional neurologic outcomes.
